# The uplift of the Qinghai–Tibet Plateau and glacial oscillations triggered the diversification of *Tetraogallus* (Galliformes, Phasianidae)

**DOI:** 10.1002/ece3.6008

**Published:** 2020-01-22

**Authors:** Li Ding, Jicheng Liao, Naifa Liu

**Affiliations:** ^1^ School of Life Sciences Lanzhou University Lanzhou China

**Keywords:** diversification, introgression hybridization, Qinghai‐Tibet Plateau, speciation, *Tetraogallus*

## Abstract

The Qinghai–Tibet Plateau (QTP) plays an important role in avian diversification. To reveal the relationship between the QTP uplift and avian diversification since the Late Cenozoic, here, we analyzed the phylogenetic relationship and biogeographical pattern of the genus *Tetraogallus* (Galliformes, Phasianidae) and the probable factors of speciation in the period of the QTP uplift inferred from concatenated data of four nuclear and five mitochondrial genes using the method of the Bayesian inference. Phylogenetic analysis indicated that *T. himalayensis* had a close relationship with *T. altaicus* and conflicted with the previous taxonomy of dark‐bellied and white‐bellied groups. The molecular clock showed that the speciation of *Tetraogallus* was profoundly affected by the uplift of the QTP and glacial oscillations. Biogeographic analysis suggested that the extant snowcocks originated from the QTP, and the QTP uplift and glacial oscillations triggered the diversification of *Tetraogallus* ancestor. Specifically, the uplift of the mountain provided a prerequisite for the colonization of snowcocks *Tetraogallus* as a result of the collision between the Indian and the Arab plates and the Eurasian plate, in which ecological isolation (the glacial and interglacial periods alternate) and geographical barrier had accelerated the *Tetraogallus* diversification process. Interestingly, we discovered hybrids between *T. tibetanus* and *T. himalayensis* for the first time and suggested that *T. tibetanus* and *T. himalayensis* hybridized after a second contact during the glacial period. Here, we proposed that the hybrid offspring was the ancestor of the *T. altaicus*. In conclusion, the uplift of QTP and glacial oscillations triggered the snowcocks colonization, and then, isolation and introgression hybridization promoted diversification.

## Graphical abstract

A phylochronogy of the genus *Tetraogallus* species based on concatenated nucleotide data. Phylogenetic analysis indicated that *T. himalayensis* had a close relationship with *T. altaicus* and conflicted with the previous taxonomy of dark‐bellied and white‐bellied groups. The molecular clock showed that the speciation of *Tetraogallus* was profoundly affected by the uplift of the QTP and glacial oscillations.

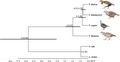

## 1 INTRODUCTION

The uplift of Qinghai–Tibet Plateau (QTP) profoundly impacts speciation and diversification in the plateau birds (Lei, Qu, & Song, 2014). The uplift of the QTP results from the collision of the Indian Plate with Eurasia during the Eocene (55–50 million years ago [Mya]) and then has experienced different stages of growth to reach the current altitude, which has created the unique topography, complex climate, and diversified habitats of the QTP, and makes the QTP an area of worldwide importance for biodiversity (An et al., 2006; Favre et al., 2015; Li & Fang, 1999). Moreover, the unique geomorphological configuration provides multitudinous refugia for animals to survive in harsh environments during the series of major ice ages of the Quaternary (2.4 Mya to the present) (Hewitt, 2000; Lei et al., 2014). Most of the currently endemic birds of the QTP have went through speciation since the Late Cenozoic in response to the QTP uplift, such as partridge *Perdix* (Bao et al., 2010), monal‐partridge *Tetraophasis* (Wen & Liu, 2010), and ground tit *Pseudopodoces humilis* (James et al., 2003). Therefore, the study on *Tetraogallus* diversification in the QTP can help reveal the geographic history of the QTP and the speciation mechanism. Snowcocks inhabit and evolve in the high‐altitude mountain land (Liu, 1998; Shen & Wang, 1963), and make it an ideal animal to investigate the relationship between geological history and speciation. However, the diversification of *Tetraogallus* has long been ignored and its biogeographical history is poorly understood so far.

**Figure 1:**
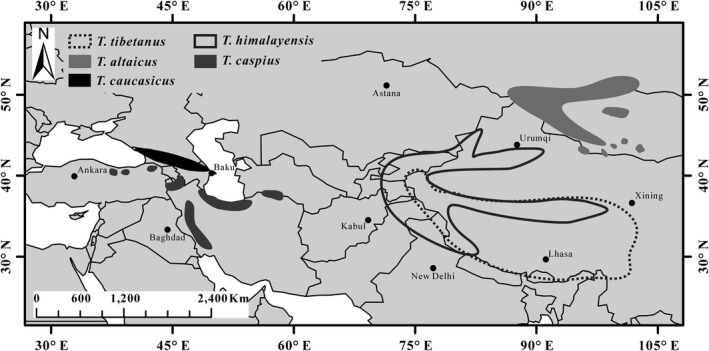
Geographical distribution of *Tetraogallus* species. Species distribution information is cited from Liu (1998)

The genus *Tetraogallus* is composed of five species, including *T. tibetanus*, *T. himalayensis*, *T. altaicus*, *T. caspius*, and *T. caucasicus*, and distributed in the major mountains system of the Center Asia (Figure 1) (Liu, 1998; Zheng, 2002). *T. tibetanus*, an endemic to the QTP, live across almost mountain systems on the whole QTP, including Pamir Plateau (Liu, 1998; Zheng, Tan, Lu, & Tang, 1978). *T. himalayensis* inhabit the Tien Shan, Pamir Plateau, and parts of the QTP, and the distribution area overlaps with *T. tibetanus* on the QTP (Liu, 1998; Zheng et al., 1978). *T. altaicus* are distributed in the Altai–Sayan–Hangay Mountains (Liu, 1998; Potapov, 1987; Zabelin, 2007), including the Altai Mountains in China (Huang, Mi, & Shao, 1992). *T. caspius* are distributed in the southern part of the Caspian Sea from the eastern Anatolian Plateau to the Iranian Plateau, and *T. caucasicus* only survive in the Caucasus Mountains (Dementiev & Gladkov, 1967; Liu, 1998). Bianki (1898) once thought that extant five snowcocks can classify into dark‐bellied (*T. himalayensis*, *T. caspius*, and *T. caucasicus*) and white‐bellied (*T. tibetanus* and *T. altaicus*) groups based mainly on adult abdomen plumage color. Liu (1998) suggested that *T. tibetanus* and *T. himalayensis* first split from the *Tetraogallus* ancestor based on their morphological characteristics and represent white‐bellied and white‐bellied groups. *T. altaicus* split from *T. tibetanus*; both *T. caspius* and *T. caucasicus* split from *T. himalayensis* and speculated that *T. altaicus* is likely to hybridize with *T. himalayensis* (Liu, 1998). Liu (1998) systematically elaborated the phylogenetic relationship of *Tetraogallus*, but the aforementioned conclusion has not been validated by molecular phylogenetic approach up to now. Stein, Brown, and Mooers (2015) reconstructed the phylogenetic relationship of *Tetraogallus* except for *T. caucasicus* using molecular data and the result indicated that *T. altaicus* has a close relationship with *T. caspius*; however, this result did not explain the morphological characteristics and the distribution pattern of extant snowcocks. Thus, a further phylogenetic study on the genus *Tetraogallus* remains critical to understand the speciation in relation to the geographic history.

The origin of *Tetraogallus* has been controversial over the past few decades (Liu, 1998). In the aspect of origin time of the *Tetraogallus* ancestor, Koslova (1952) and Baziev (1978) suggested that snowcocks originate in the Early Pleistocene (about 2.5 Mya) based on the orogeneses of the QTP. Potapov (1992) disagreed with the aforementioned point and proposed that the ancestor of *Tetraogallus* occurs in the first glacier of the Pleistocene (Minde glacial period) because the climate of the Pliocene/Pleistocene boundary is warm and does not have the conditions for the origin of the *Tetraogallus*, but that is too late for snowcocks to have evolved obviously. As such, Liu (1998) pointed out that the snowcock first occurs in the Quaternary Early glaciation (Hongya glaciation, about 3.5–2.6 Mya). However, recent molecular phylogenetic studies have greatly advanced the origin of snowcocks. Stein et al. (2015) suggested that the ancestor of *Tetraogallus* can be dated back to the Middle Oligocene (about 29.32 Mya) and diversification begins in the Late Miocene (about 7.31 Mya) inferred from the concatenation dataset of nuclear and mitochondrial genes. In the aspect of origin area of the *Tetraogallus* ancestor, Koslova (1952) pointed out that the origin of the snowcock‐like ancestors is related to the uplift of the QTP, and they may be originated from the jungles of the Eastern Kunlun Mountains and the Hengduan Mountains (Western Sichuan Province, China). Baziev (1978) believed that the snowcock‐like ancestor is not alpine birds, and they inhabit the hilly areas from the Caucasus to Central China; the snowcock‐like ancestor will rise with the process of mountain uplift, so each mountain system has a kind of snowcock. Consequently, the distribution area of extant snowcocks is the origin of ancestor. However, Potapov (1992) found a paradox that snowcocks are unlikely to inhabit the Hengduan Mountains during the Pliocene/Pleistocene boundary because of lower altitude and suggest that snowcocks originated in the Pamir Plateau, Tien Shan, and Kunlun mountains. Fossil data can be used to estimate the origin of snowcocks, in addition, however snowcocks have few fossils available, and precious fossil data are only found in the Altai and Caucasus Mountains, which can date back to the Middle and Late Pleistocene, and these are not sufficient to infer the origin of *Tetraogallus* because time is too short (Panteleev, 2002; Potapov, 1992). In summary, the origin of the *Tetraogallus* ancestor has not yet been determined in terms of spatial and temporal.

To gain knowledge on the speciation mechanisms in snowcocks, here, we investigated the phylogeny, biogeographical history, and diversification rate using a concatenated DNA dataset. As a consequence, the present investigation aimed at solving three questions: 1. When and where did snowcocks originate? 2. Was the diversification of snowcocks affected by the QTP uplift and glacial oscillations? 3. Was hybridization promoting the speciation of *T. altaicus*?

## 2 MATERIALS AND METHODS

### 2.1 Taxon set and DNA data

The genus *Tetraogallus* is composed of five species (*T. himalayensis*, *T. tibetanus*, *T. altaicus*, *T. caspius*, and *T. caucasicus*) according to taxonomic data, which belong to Galliformes, Phasianidae (Zheng, 2002). Here, *T. caucasicus* was not taken into account in the ingroup taxon set because its DNA data were not available up to now. All DNA sequence data were downloaded from the GenBank database (downloaded on or before December 30, 2018; Table A1) in the present study. Two outgroup taxa were selected from the genus *Alectoris* based on the previous study (Stein et al., 2015), including *A. chukar* and *A. rufa*. We assembled a DNA data matrix which was composed of 3 protein‐coding genes (COX1, CYTB, and ND2) and 2 nonprotein‐coding genes (12S and D‐loop) mitochondrial loci combined with 4 nonprotein‐coding genes (CLTC, CLTCL1, RHO, and EEF2) nuclear loci (Table A1). The DNA matrix was 6,975 base pairs (bp) long at its maximum extent (including gaps), and the alignment data were sparse and average coverage was approximately 85% across DNA markers (mitochondrial locus: 67%–100%; nuclear locus: 83%). In order to improve the resolution ability of the phylogenetic inference, here, we chose to mark as high coverage as possible rather than adopted all publicly available nucleotide sequences. In addition, D‐loop sequences were downloaded from the GenBank database and were used to construct phylogenetic analysis of populations between *T. tibetanus* and *T. himalayensis*. Sequence information please see Table A2.

### 2.2 Phylogenetic analysis, genetic distance, nucleotide mutation rate, and estimation of divergence time

Multiple‐sequence alignments of mitochondrial and nuclear genes were performed using BioEdit v. 7.1.3.0 program (Hall, 1999) with default parameters. Each gene was aligned separately and manually concatenated these sequences using Sequence Matrix v. 1.7.8 (Vaidya, Lohman, & Meier, 2011). All concatenated sequences were format converted for further analyses using Geneious v. 9.1.4 program. The genetic distance was calculated by MEGA 6.0 (Tamura, Stecher, Peterson, Filipski, & Kumar, 2013) with the Kimura 2‐parameter (K2P) model of nucleotide substitution (Kimura, 1980) using concatenated data. Phylogenetic analysis and divergence date on the genus *Tetraogallus* species were estimated by BEAST version 1.7.4 program (Drummond & Rambaut, 2007) using the Markov Chain Monte Carlo (MCMC) to estimate the divergence times of the genus *Tetraogallus*. The best‐fit model of substitution and best partition schemes for the dataset were identified with the corrected Akaike information criterion (AICc) (Akaike, 1974), implemented in Modeltest 3.7 program (Posada & Crandall, 1998).

To acquire as accurate divergence time as possible, we estimated the number of nucleotide substitutions per site (*d*) from comparisons of the focal species and an outgroup species using the formula: *d* = (*tv* + *tvR*)/*m*, where *tv* is the number of transversions between the genus *Tetraogallus* and outgroup taxa, *R* is the transition/transversion ratio within the genus *Tetraogallus*, and *m* is the sequence length (Nei, 1992; Rooney, Honeycutt, & Derr, 2001). Transition and transversion values were calculated in the program MEGA 6.0. The rate of nucleotide substitutions per site per lineage per year is *λ* = *d*/2*T* when an estimate of *d* was obtained, where *T* is the divergence time between the ingroup and outgroup species (Rooney et al., 2001). The mutation rate per nucleotide site per generation is *μ* = *λg*, where g is the generation time (*g* = 3 years of snowcock; Huang, Ma, Shao, & Jiang, 1990). Here, *d* was 0.084 (*tv* = 84, *R* = 5.96, *m* = 6,975) for combined genes. The rate of nucleotide substitution per site per lineage per year (*λ*) was about 0.14 × 10^–8^ (*T* = 29.32 Mya, quoted from Stein et al. (2015)), and the mutation rate of per generation (*μ*) was about 0.40% Mya for concatenation DNA sequence of snowcocks. Similarly, we used the same method to calculate the rate of nucleotide substitute of D‐loop and the result showed that *μ* was about 0.20% Mya.

The relaxed molecular clock was performed with the uncorrelated lognormal clock model for species divergence of *Tetraogallus* to infer branch lengths and nodal ages under the GTR + I substitution model in BEAST. The phylogenetic relationship was reconstructed under the Yule speciation process (Steel & McKenzie, 2001), with enforced monophyly of the ingroup. Chain lengths were 50 million, sampled every 1,000 generations, with the first 10% discarded as burn‐in. The posterior distribution of the estimated divergence times was obtained by specifying one calibration point that was used as prior for the time for the most recent common ancestor of *A. chukar* and *A. rufa*. According to the previous studies, the age of the *A. chukar* and *A. rufa* divergence was as time calibration point (about 2.84 Mya) (Stein et al., 2015). The prior for the age of the tree root was corresponding to the basal split, and a normal distribution was as the prior model for the calibration with mean 2.84 Mya and standard deviation 0.01. As such, the similar setting in BEAST was used to estimate the population divergence date between *T. tibetanus* (35 haplotypes; An, Zhang, Liu, & Wang, 2015) and *T. himalayensis* (37 haplotypes; Wang, Qu, Liu, Bao, & Song, 2011) with the Bayesian strict clock under the HKY + I + G substitution model. The final molecular clock tree was generated in the program TreeAnnotator 1.7.4 (a subprogram of BEAST) using the mean as node heights. The program Tracer v1.5 was used to test the results validity of sampling (effective sample size (ESS) > 200). Here, Bayesian posterior probability (PP) equal or above 0.95 was considered as strong relationships (Leaché & Reeder, 2002). Finally, we used the FigTree v. 1.3.1 program to visualize all tree files.

### 2.3 Biogeographical analysis

To infer the possible origin area of the genus *Tetraogallus*, we used the program with the method of BBM (Bayesian binary MCMC) in RASP (Reconstruct Ancestral State in Phylogenies) (Yu, Harris, Blair, & He, 2015) to reconstruct the possible ancestral region of snowcocks on the phylogenetic tree. Here, the biogeographic distribution range of extant species of *Tetraogallus* was divided into four sections according to the available literature (Liu, 1998; Potapov, 1987; Zheng et al., 1978) and the International Union for Conservation of Nature online source (IUCN, https://www.iucnredlist.org). These areas were A (Qinghai–Tibet Plateau [including Pamir Plateau]), B (Tien Shan Mountains), C (Altai–Sayan–Hangay Mountains), and D (Iranian–Anatolia Plateau) (Figure 3a, Top‐left). We used a phylogenetic tree of *Tetraogallus* that derived from BEAST analysis output and ran BBM on all of them, and the number of maximum areas was set to 1 area. The MCMC chains were run five millions generation and sampled every 100 generations. Fixed JC + G (Jukes–Cantor + gamma) was used for BBM analysis with null root distribution. The possible ancestral ranges were determined at each node on a selected tree.

### 2.4 Diversification analysis

To reconstruct the macroevolutionary dynamics of *Tetraogallus* over time, we implemented a diversification rate analysis. BAMM software used a Bayesian framework to account for rate variation through time and among lineages in phylogenetic trees, and we employed it to simulate posterior distributions of rate‐shift configurations (Rabosky, Donnellan, Grundler, & Lovette, 2014a). We then used BAMMtools package in R to conduct rate‐through‐time analysis and to identify and visualize diversification rate shift along branches (the BEAST output result) (R Core Team, 2012; Rabosky, Grundler, et al., 2014b). BAMM was run by setting four independent MCMC running for 10 million generations and sampled every 1,000 generations. After removing 25% of trees as burn‐in, the BAMM output was analyzed with employing BAMMtools and the 95% credible rate‐shift configurations were estimated using Bayes factors. The best shift configuration with the highest maximum a posteriori probability was estimated in this analysis. In addition, we demonstrated a relationship between diversification rate shifts and ecological opportunity processes, which if ecological opportunity occurred in new regions could trigger rapid radiation and cause speciation rate increase and colonization of a new region (Schenk, Rowe, & Steppan, 2013; Yoder et al., 2010). The phytools package in R was used to estimate a gamma statistic (*γ*) with a phylogenetic tree to test region‐specific patterns in the diversification rates and to visualize lineage‐through‐time (LTT) plot (Revell, 2012). Herein, if *γ* value was positive (*γ* > 0), it suggested a late outbreak of speciation rate, and conversely, a negative value for *γ* (*γ* < 0) indicated that this branch has a process of decreasing the speciation rate (Pybus & Harvey, 2000).

## 3 RESULTS

### 3.1 Phylogenetic analysis, divergence time, and genetic distance

**Figure 2:**
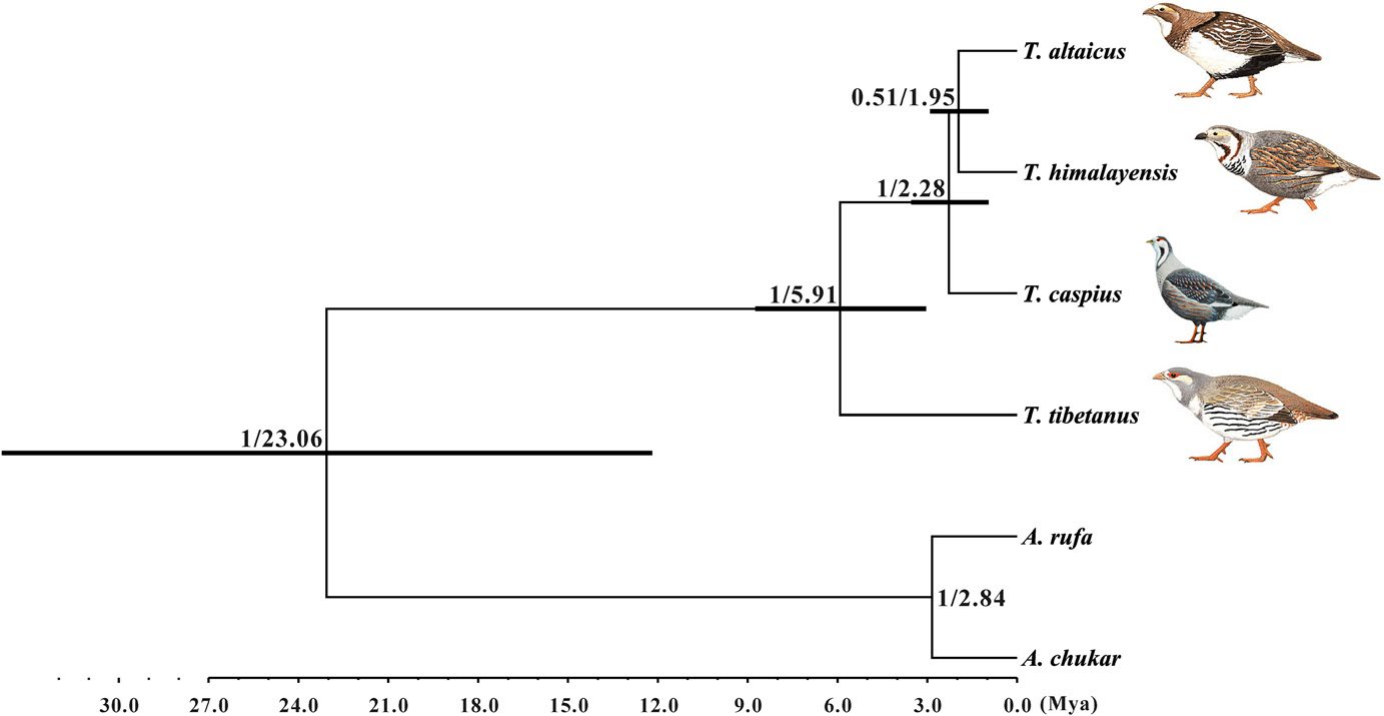
A phylochronogy of the genus *Tetraogallus* species based on concatenated nucleotide data. The divergence time is estimated using the BEAST with the calibration method under the relaxed molecular clock model (using the estimated mutation rate). Branch lengths represent the mean values of the posterior distribution. The posterior probability and divergence time are indicated at each inner node. The node bars indicate the posterior probability distribution of the node age under the 95% CI. Snowcock portraits are quoted from MacKinnon, Phillipps, He, and Lu (2000) and Svensson, Mullarney, Zetterstrom, and Grant (2009)

Phylogenetic relationships of the genus *Tetraogallus* were reconstructed using the concatenated DNA data with the method of Bayesian inference. The results indicated that *T. tibetanus* and *T. himalayensis* were first split from the *Tetraogallus* ancestor (PP = 1) and dated back to the Late Miocene (about 5.91 Mya) with 95% confidence intervals (95% CI) of 3.09–8.87 Mya (Figure 2). *T. caspius* split from *T. himalayensis* (PP = 1) that occurred in the Early Pleistocene (about 2.28 Mya, 95% CI: 1.10–3.66 Mya). *T. altaicus* was closely related to *T. himalayensis* by comparison with *T. tibetanus* (PP = 0.51) and split from *T. himalayensis* that first occurred in the Early‐Middle Pleistocene (about 1.95 Mya, 95% CI: 0.98–2.92 Mya). The K2P distance between snowcocks was calculated using concatenated data (Table 1). The *T. tibetanus* showed the high divergence (between 0.060 and 0.064), with the most divergent lineages differing by K2P distance inferred from the concatenated data. However, the K2P distance between *T. himalayensis* and *T. altaicus* was quite low (K2P = 0.007) by comparisons with other snowcocks, implicating the lower differentiation between them in line with the phylogeny tree (Table 1, Figure 2).

**Table 1:** Pairwise distances divergence (K2P distances) for the concatenated DNA data between the genus *Tetraogallus* species

	*T. altaicus*	*T. caspius*	*T. himalayensis*	*T. tibetanus*
*T. altaicus*				
*T. caspius*	0.017			
*T. himalayensis*	0.007	0.013		
*T. tibetanus*	0.061	0.064	0.060	

### 3.2 Biogeographical analysis

**Figure 3:**
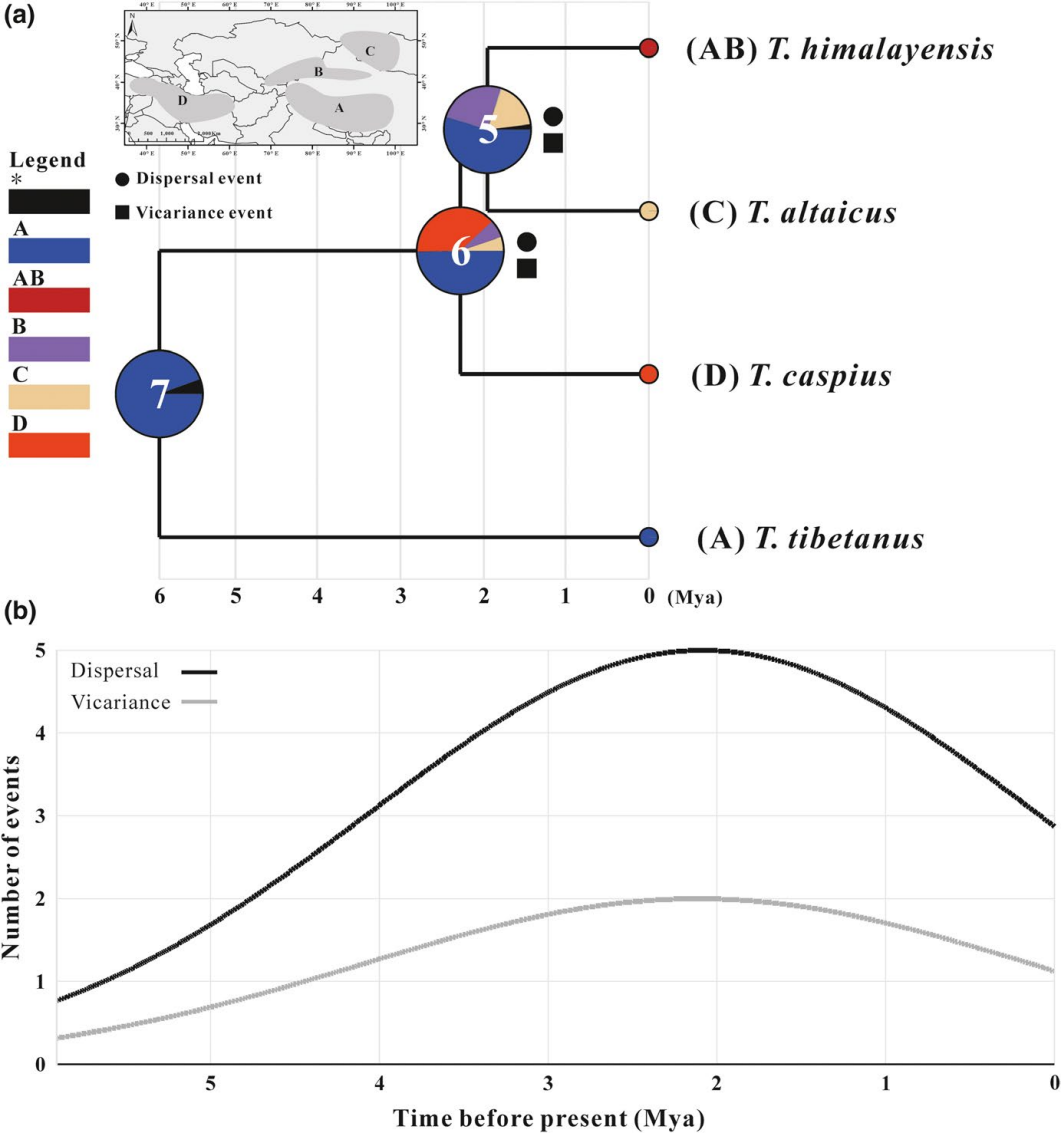
Diagram of ancestral distribution ranges is estimated using BBM analysis (output from RASP). (a) Biogeographical areas used in RASP analysis are divided into four regions (Top‐left). The possible ancestral range is estimated by BBM based on each node of the phylogeny of the genus *Tetraogallus*, and Pie charts at each node show probabilities of alternative ancestral ranges. (b) The frequency of biogeographic events of snowcocks changes over time

BBM analysis indicated that the ancestor of the genus *Tetraogallus* originated from the area A at node 7 with high marginal probability (94.4%) (Figure 3a). Similarly, the ancestral reconstruction of BBM suggested that A was the ancestral area of nodes 6 and 5, with 50.1% and 55.1% marginal probability, respectively. BBM results suggested that the speciation between *T. tibetanus* and *T. himalayensis* occurred in the QTP during the Late Miocene (node 7), and then they underwent dispersal and isolation that may be driven by the geographic and climatic events, as evident from ancestral ranges at nodes 6 and 5. BBM detected two vicariance events and three dispersal events at nodes 6 and 5, while node 7 without any biogeographic event occurred. Moreover, BBM estimated the possible colonization route of snowcocks (nodes 7, 6, and 5) as the result that the origin and spread of snowcocks were related to the QTP uplift. In addition, the time curve of dispersal and vicariance events illustrated that a fastigium of biogeographical events occurred in the Early Pleistocene (about 2.28 Mya) (Figure 3b). Herein, it was clear that this discontinuous distribution pattern was related to geological and climatic changes.

### 3.3 Diversification rate shift

**Figure 4:**
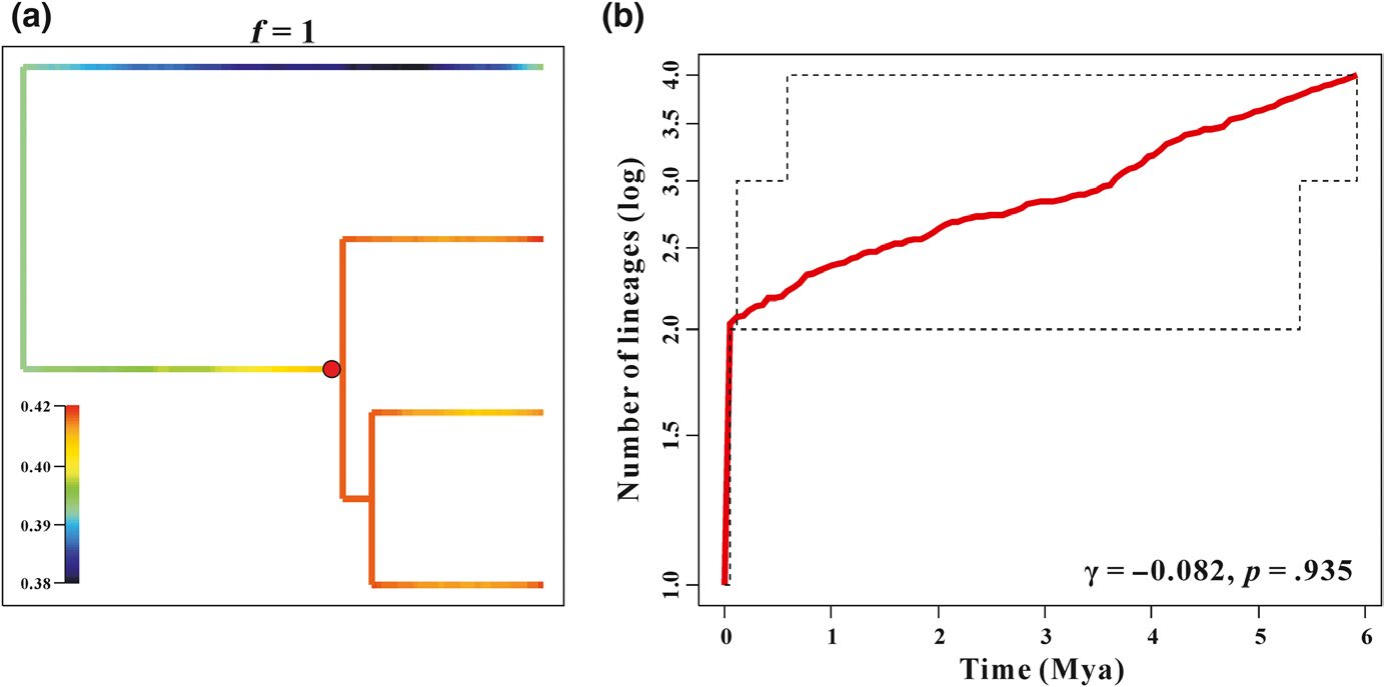
Diagram of macroevolutionary dynamics of *Tetraogallus*. (a) A phylorate plot shows speciation rates (cool colors = slow and warm = fast) along each branch of the *Tetraogallus* phylogeny. The red dot denotes the position of the diversification rate shift detected by BAMM analysis. (b) LTT plot of *Tetraogallus*. The red solid line shows the mean number of lineages and the black dashed lines along with it give the 95% confidence interval

The distinct shift configuration for diversification was shown in Figure 4a, along with the corresponding consensus phylorate plot and rate acceleration events within the genus *Tetraogallus* taxa which fell into the Early Pleistocene (about 2.28 Mya). Here, the speciation rate of snowcocks has undergone a rapid increase and then subsequent decrease, and we detected the best rate‐shift point which was noted in the phylorate plot (Figure 4a). The LTT plot showed an increase of diversification rate after colonized the QTP area (Figure 4b) and suggested a potential early initial burst at approximately 6 Mya, and after that the speciation rate has undergone a subsequent reduction (*γ* = −0.082, *p* > .05) in line with phylorate (Figure 4a).

### 3.4 Phylogeographic analysis between *T. tibetanus* and *T. himalayensis*

**Figure 5:**
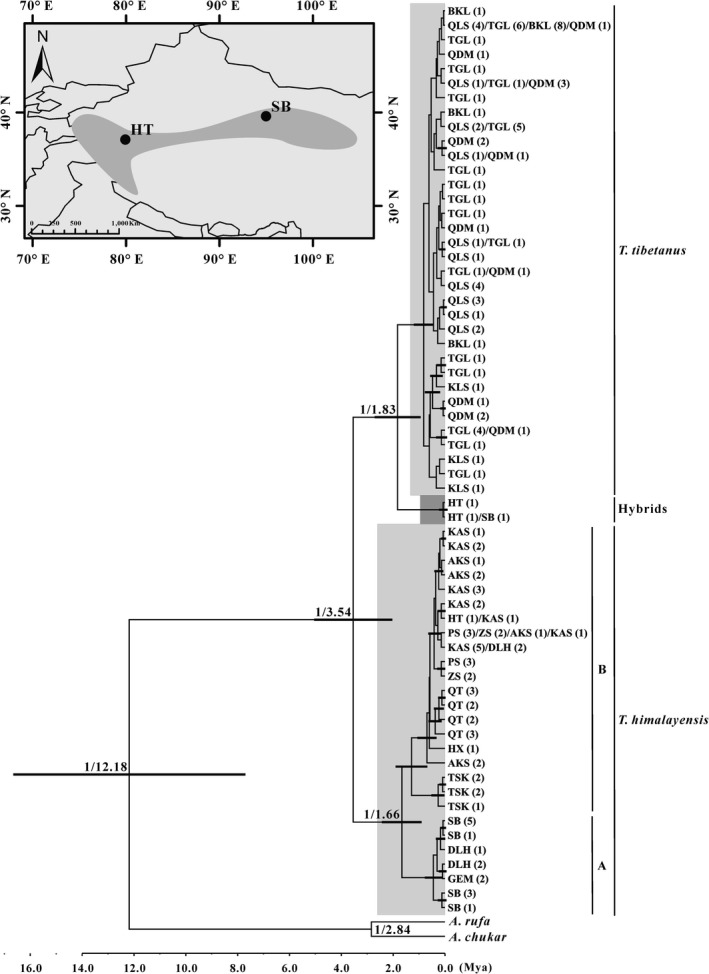
A chronogram of population divergence between *T. tibetanus* and *T. himalayensis* based on D‐loop haplotypes. The divergence time is estimated using the BEAST with the calibration method under the strict clock model (using the estimated mutation rate). Branch lengths represent the mean values of the posterior distribution. The posterior probability and divergence time are indicated at the major inner nodes, and population names are provided in Table A2. The node bars indicate the posterior probability distribution of the node age under the 95% CI. Proposed hybridization zone of *T. tibetanus* and *T. himalayensis* is marked in dim gray shade (Top‐left)

Phylochronology showed two distinct lineages (PP = 1) and the population divergence time between *T. tibetanus* and *T. himalayensis* occurred in the Middle Pliocene (about 3.54 Mya) with 95% confidence intervals of 2.19–5.20 Mya. There, no phylogeographic structure was observed with *T. tibetanus* and all haplotypes were intermixed with poor support and low divergence (Figure 5) and suggested that there were frequent genetic exchanges between populations. All haplotypes of *T. himalayensis* grouped into two clades (Clade A and Clade B) with high support value (PP = 1); Clade A represented the populations from the Northern Qinghai–Tibet Plateau and Clade B represented the populations from the Kunlun–Tien Shan mountains. The divergence time between two major clades dated back to the Early Pleistocene (about 1.66 Mya, 95% CI: 0.99–2.52 Mya). More importantly, we found three hybrids between *T. tibetanus* and *T. himalayensis* from the SB and HT populations (Figure 5, dark gray clade). Three hybrids should be *T. himalayensis* but have a close relationship with *T. tibetanus* (PP = 1), indicating that the male *T. himalayensis* hybridized with the female *T. tibetanus* as the result that hybrids embed into *T. tibetanus* clade. The molecular clock indicated that hybridization time between *T. himalayensis* and *T. tibetanus* occurred in the Early Pleistocene (about 1.83 Mya, 95% CI: 1.04–2.81 Mya). Here, we speculated that the hybridization area was likely to be located on the sympatric zone of *T. tibetanus* and *T. himalayensis* (Figure 5; Top‐left: dim gray shade).

## 4 DISCUSSION

A multi‐locus phylogeny indicated that *T. tibetanus* and *T. himalayensis* first differentiated from the *Tetraogallus* ancestor, and then *T. altaicus* and *T. caspius* split from *T. himalayensis* (Figure 2), and our results supported the conclusions proposed by Liu (1998). Phylogenetic analysis suggested that *T. himalayensis* has a close relationship with *T. altaicus* by comparison with *T. caspius* which vary from previous phylogenetic result (Stein et al., 2015), and we believed the current results were rational because of the large number of phylogenetic information sites provided. As such, the K2P distance also supported this result (Table 1). Stein et al. (2015) suggested that *T. altaicus* has close relationship with *T. caspius,* but their work cannot explain the speciation mechanism because of the distribution pattern of extant snowcocks and morphological variations. Both *T. tibetanus* and *T. altaicus* are classified into white‐bellied group (Bianki, 1898), at this point, but *T. altaicus* was closely related to *T. himalayensis* in terms of morphological and genetic characteristics in fact rather than *T. tibetanus*. Phylogenetic results on the genus *Tetraogallus* broke the knowledge of white‐bellied and dark‐bellied groups by taxonomists for a century, making this conclusion be challenged. We would discuss how extant snowcocks evolved below. The divergence time of calibration point indicated that our time was basically consistent with Stein et al. (2015)' results and suggested that the ingroup divergence time was rational. The speciation event between snowcocks can be dated back to the Late Miocene to Early Pleistocene (5.91–1.95 Mya) which predated previous studies (Baziev, 1978; Koslova, 1952; Liu, 1998; Potapov, 1992) and can adequately reveal the speciation mechanism of snowcocks because for this timeframe takes about 2 million years for it a vertebrate to evolve into a new species (Avise, Walker, & Johns, 1998). More importantly, the speciation of snowcocks coincided with the uplift of the QTP and the Quaternary glacial oscillations. For instance, Bao et al. (2010) suggested that the speciation of the genus *Perdix* is triggered by the Late Pliocene/Early Pleistocene (3.63–2.00 Mya) intensive uplift of the QTP and the Pleistocene glaciations (2.05–1.28 Mya). Naturally, we proposed that the *Tetraogallus* diversification was affected by the uplift of the QTP and the Quaternary glacial period at that time.

BBM analysis indicated that the *Tetraogallus* ancestor originated from the QTP (including Pamir Plateau) (Figure 3). Previous studies on the origin area of snowcocks supported our result (Koslova, 1952; Liu, 1998; Potapov, 1992) and suggested that the uplift of the QTP triggered the *Tetraogallus* diversification. The uplift of the QTP is a complicated geological development process and has underwent several uplifts which is the progressive and heterogeneous uplift of the QTP from south to north (Favre et al., 2015; Mulch & Chamberlain, 2006; Wang et al., 2008). Subsequently, a series of rapid uplift events give rise to approach its present elevation during the Late Cenozoic (Fang, 2017; Li & Fang, 1999; Shi, Li, & Li, 1998), including Qing–Zang (between 3.6 and 1.7 Mya), Kunlun–Huanghe (between 1.1 and 0.6 Mya), and Gonghe (about 0.15 Mya) tectonic movements. Specifically, the Qing–Zang tectonic movement occurred in the Middle Pliocene to Early Pleistocene (between 3.6 and 1.7 Mya), which contained three phases A (begin in 3.6 Mya), B (2.5 Mya), and C (1.7 Mya) (Li & Fang, 1999). The progressive extension of the uplift of the QTP is associated with the major mountains uplift and has blocked the northward of the India Ocean warm air, such as the higher Himalayas and the rise of the Tien Shan, which causes the progressive aridification of Central Asia during the Miocene (Miao, Herrmann, Wu, Yan, & Yang, 2012; Miao et al., 2011; Sun, Gong, Tian, Jia, & Windley, 2015). Moreover, the development of the Pleistocene glaciers of the QTP is closely related to the progressive uplift of the plateau and the surrounding mountains (Zheng & Rutter, 1998). Consequently, the QTP after the uplift results in drastic shifts in the distribution of plant communities and major faunal turnover (Deng & Ding, 2015; Li, Fang, Pan, Zhao, & Song, 2001; Sun & Wang, 2005). All these factors can be beneficial snowcocks to acquire suitable ecological resource and promoted adaptive radiation, of which the geographic colonization is an important way to survive (Stroud & Losos, 2016). The colonization of novel territory (e.g., mountains uplift or glacier recession) can provide a release from competition and predation pressures, abundant food resources, or suitable climatic conditions, allowing them to differentiate a variety of species to colonize multiple unexploited ecological resources, such as ground tit (James et al., 2003) and voles (Lv, Xia, Ge, Wu, & Yang, 2016). In summary, this evolutionary model can be characterized by ecological opportunity. Ecological opportunity can affect speciation rate, and animals will soon fill unoccupied niche if rate increase and then decrease tend to be stable finally (Rabosky & Lovette, 2008; Schenk et al., 2013). These theories were in favor of the diversification mechanism of snowcocks. Here, the diversification analysis of snowcocks indicated that the increase of speciation rate can be dated back to the Late Miocene and peaked in the Early Pleistocene (about 2.28 Mya, Figure 4), which coincided with the active period of the Late Cenozoic of the QTP. Obviously, the phases of A and B profoundly influenced on the snowcocks speciation and supported the snowcocks speciation was subjected to ecological opportunity. As such, the LTT analysis showed that the snowcocks speciation was subjected to ecological opportunity even though the gamma value was not significant because of the small number of species. In conclusion, the uplift of the QTP provided ecological opportunity for *Tetraogallus* diversification.

The *T. tibetanus*, an endemic to the QTP, has the long evolutionary history among the extant snowcocks. *T. tibetanus* split from *T. himalayensis* that can date back to the Late Miocene to coincide with the geological active period of QTP and Asian interior aridification (Sun et al., 2015), namely that the uplift of the QTP promoted speciation of *T. tibetanus*. Here, similar geological event occurred on the *Phrynocephalus theobaldi* differentiation (about 5.65 Mya), and the plateau uplift promoted the differentiation of toad‐headed lizards that inhabited the QTP during the Late Miocene (Jin, Liu, & Brown, 2017), and maybe, similar geographic events induced the divergent possibility between *T. tibetanus* and *T. himalayensis* at that time. Theoretically, the Gloger's rule believes that melanin is abundant in hot and humid areas, while maroon pigment or tawny pigment is abundant in dry areas (Edward, Burtt, & Jann, 2004; Zheng, 1952). We speculated that the origin area of *T. tibetanus* was likely to occur in the Gangdise Mountains and the Himalayas with wetter air and warm climate before the intense uplift of the QTP during the Late Cenozoic (since 3.6 Mya, Li and Fang (1999)) based on black stripes of belly feathers. Moreover, the origin area of *T. himalayensis* was likely to take place in the Pamir Plateau or the area east of the Pamir Plateau (Western Kunlun Mountains) with drier air and cold climate. A rational explain was that the Gangdise and Himalayas are the first to uplift as the result that has caused ecological shift in different regions and can provide ecological opportunity for *T. tibetanus* and *T. himalayensis* to colonize because the geographical development of the QTP is progressive uplift from south to north (Li & Fang, 1999; Mulch & Chamberlain, 2006). Competitive exclusion and ecological isolation caused the differentiation between *T. tibetanus* and *T. himalayensis* (Liu, 1998, 1999). Ecological opportunity had created a novel habitat for *T. tibetanus* and *T. himalayensis*, and then adaptive radiation accelerated them to divergence. Consequently, ecological isolation drove two snowcocks (*T. tibetanus* and *T. himalayensis*) to occupy different niches to evolve in their respective directions (Liu, 1999), including altitude, diet, breeding strategy, and breeding time, and finally evolved into two separate species. Geographical or ecological isolation leads to a reduction or disruption of genetic exchange between populations, and alleles change under the influence of genetic drift, which ultimately give rise to reproductive isolation (Hoskin, Higgie, McDonald, & Moritz, 2005; Via, 2012). For example, the uplift of the QTP gives rise to the speciation of the genus *Perdix* (Bao et al., 2010), and ecological divergent is an important factor for eared pheasants *Crossoptilon* speciation (Wang et al., 2017). In summary, the uplift of the QTP and niche divergence promoted the speciation of *T. tibetanus* and *T. himalayensis*.

Liu (1998) suggested that snowcocks colonize a novel habitat during the glaciation period, and then, interglacial period isolation promotes speciation. *T. caspius* split from *T. himalayensis* can be dated to the Early Pleistocene (about 2.28 Mya) corresponding to the uplift of the QTP and the Quaternary glacial oscillations and suggested that *T. himalayensis* colonized to the Iranian Plateau and Anatolia Plateau during the ice age and interglacial period isolation forced the independent evolution of the dispersed populations to eventually evolve into *T. caspius*. It was worth noting that the dispersal of *T. himalayensis* reached a climax during this period (about 2.28 Mya) inferred from BBM analysis. The Iranian Plateau is a juvenile landmass, which begins to uplift as a result of the Arabian plate impinging on Eurasia during the Late Eocene to the Early Miocene (between 35 and 20 Mya) (Berberian, 2014; Mouthereau, 2011). After that, the tectonic movements of Iranian Plateau give rise to the climatic change and the formation extensions of steppe‐like landscape during the Middle and Late Miocene (Storch, 2004), in which provides several suitable habitats for snowcock survival and also provides a prerequisite for *T. himalayensis* colonization. Furthermore, the Northern hemisphere great glacial period has led to the widespread development of ice sheets or glaciers in low‐latitude areas (An et al., 1998; Curry, 1966; Wu & Li, 1990) and promoted the *T. himalayensis* to step out the Pamir Plateau to colonize the Iranian Plateau, Anatolia Plateau, and Caucasus Mountains. Interglacial period isolation caused the speciation of *T. caspius*. BBM analyses detected vicariance and dispersal events in the speciation proceed of *T. caspius* (Figure 3) and supported this interpretation.

**Figure 6:**
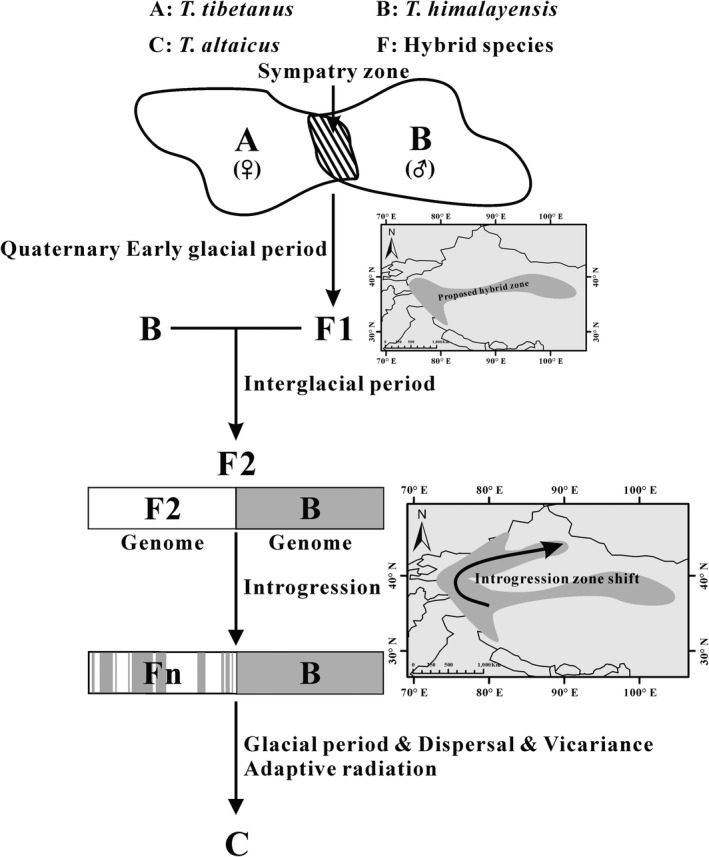
Proposed speciation model of *T. altaicus*. The hybridization between *T. tibetanus* and *T. himalayensis* occurs in the sympatric zone during the Quaternary Early glacial period, and then reproduces F1 generation. The F1 hybridizes with *T. himalayensis* and reproduces F2 during the interglacial period, and then several generations of introgression allowed hybrids to inherit numerous loci from *T. himalayensis*, which can be combined via gene flow through hybridization. Competitive exclusion between them gives rise to the introgression zone shift along with the Tien Shan and makes Fn (hybrids) to be an ancestor for *T. altaicus*. Hybrids (Fn generations) can potentially be adaptive and favored in a new habitat via adaptive introgression and can lead to a separate hybrid taxon (hybrid speciation). Finally, competitive exclusion and ecological opportunity can explain that hybrid species (the ancestor of *T. altaicus*) colonize the Altai–Sayan–Hangay Mountains during the glacial period, and then, geographical isolation and adaptive radiation have driven hybrids to be a novel species—*T. altaicus*

Phylogeographic analysis results indicated that three hybrids were reproduced by the hybridization between female *T. tibetanus* and male *T. himalayensis* based on D‐loop haplotypes (Figure 5), and they embed into the *T. tibetanus* clade because of the maternal inheritance of mitochondrial DNA (Sato & Sato, 2012). Obviously, there was no rigorous reproductive isolation between *T. tibetanus* and *T. himalayensis*, and the hybridization can give birth to F1 generation in the sympatric zone (Figure 6). Here, hybridization time can be dated back to the Early Pleistocene (about 1.83 Mya, 95% CI: 1.04–2.81 Mya). When allopatric populations meet in the contact zone, theoretically, hybridization may occur if reproductive isolation is incomplete (Dowling & Secor, 1997). When allopatric taxa became sympatric again, moreover, speciation can accelerate because of secondary contact such as the genus *Drosophila* (Coyne & Orr, 1997). For instance, the Darwin's Heath (*Coenonympha darwiniana*) originate through hybridization between the Pearly Heath (*C. arcania*) and the Alpine Heath (*C. gardetta*) with different parental contributions (Capblancq, Després, Rioux, & Mavárez, 2015), and hybridization promotes speciation in *Coenonympha* butterflies. A possible explanation is that the Quaternary Early ice age (e.g., Danube‐Gonzi ice age (about 2.6–1.5 Mya), Hongya ice age (about 2.5 Mya) (Liu, 1998), and Xixiabangma ice age (about 2.5–0.78 Mya) (Zheng, Xu, & Shen, 2002)) caused *T. tibetanus* and *T. himalayensis* to secondary contact and hybridize in sympatric zone, and then, the introgressive hybridization between F1 and *T. himalayensis* occurred in the interglacial period (Figure 6). Here, we called F2 the ghost of introgression past to be an ancestor for *T. altaicus*. Consequently, F2 repeatedly backcrosses to *T. himalayensis* as the result that a large number of alleles form *T. himalayensis* introgress into the ancestor of *T. altaicus* (Fn) (Figure 6). A further study would be to investigate introgression of *T. altaicus* from *T. tibetanus* and *T. himalayensis* at the whole genome scale. Liu (1998) pointed out *T. altaicus* have introgression from *T. himalayensis* based on morphological comparison between them, and our results have validated his speculation. Introgression hybridization (the one‐way flow of genes) between F2 and *T. himalayensis* allows *T. altaicus* to obtain parental characteristics, including morphology, behavior, and life history. The *T. altaicus* inherited many of the characteristics of the *T. himalayensis*, such as similar body size, yellow skin around the eyes, the same life habits, and tail‐hanging behavior (snowcocks portrait in Figure 2). The hybrids between *A. chukar* and *A. magna* repeatedly backcross to *A. magna*, for instance, making hybrids closely related to *A. magna* rather than that of *A. chukar* in terms of morphological and genetic characteristics (Chen, An, & Liu, 2016; Liu, Wen, Huang, & Hou, 2006), and supported our inference. The niche overlap forced introgression zone shift to colonize new suitable habitats and finally arrived in the Altai–Sayan–Hangay Mountains along the Tien Shan Mountains (Figure 6), and the ecological opportunity made them occupy new ecological resources.

Introgression hybridization usually does not generate a new species, theoretically, but if hybrids (Fn) no longer backcross with the parental population and occupy new habitats, and then introgression of a few loci may promote adaptive divergence and so facilitate speciation (Abbott et al., 2013). The differentiation time between *T. himalayensis* and *T. altaicus* occurs in the Early Pleistocene corresponding to the uplift of Pamir–Tien Shan and the Asian aridification development (Fu, Ninomiya, & Guo, 2010; Wang et al., 2014). The uplift of the Pamir–Tien Shan provided an ecological opportunity for the ancestor of *T. altaicus* to colonize a novel territory and ecological resources, and provided a prerequisite for the introgression zone shift. In addition, the uplift of the Altai–Sayan–Hangay region occurred from the Oligocene to the Pliocene and approximately reached the present landscape the end of the Early Pliocene (3.6–2.4 Mya), blocking moisture from Siberian reached rain shadow, which caused aridization in the Central Asia (Caves, Sjostrom, Mix, Winnick, & Chamberlain, 2014; Xu, Ji, Sun, & Zhao, 2015; Zabelin, 2007). The uplift of the Altai and Hangay link to India–Asia convergence corresponds to the uplift of QTP (Caves et al., 2014; Xu et al., 2015) as a result that can provide suitable habits for hybrids (Fn) survival before the speciation of *T. altaicus*, and then, they can reach the Altai–Sayan–Hangay region through the Tien Shan during the Pleistocene glacial period. The mountain land of the Tien Shan and the Altai mountains is covered by the cover‐type glacier during the glacial maxima of the Middle Pleistocene, here, make the lower limit of glacier to spread low land with the above sea level of 1000 m (Chih‐chiu, 1968). These factors provide a prerequisite for the hybridization zone shift and the dispersal of the ancestor of *T. altaicus*. Since then, the aridification of central Asia has promoted Gobi Desert development, such as Gurbantunggut Desert and Badain Jaran Desert. Geographical barriers blocked genetic exchange with *T. himalayensis*, and BBM analysis detected vicariance and dispersal events in speciation of *T. altaicus* (Figure 3a). Here, hybridization and geographical isolation accelerated the process of *T. altaicus* speciation. A few studies suggested that some birds evolved a novel species by hybridizing in natural environments. For instance, Brelsford, Mila, and Irwin (2011) suggested that the speciation of Audubon's warbler (*Dendroica auduboni*) is hybridized by myrtle warbler (*D. coronata*) and black‐fronted warbler (*D. nigrifrons*) inferred from amplified fragment length polymorphism and molecular markers data. Joseph et al. (2008) considered that the intermediate plumage phenotypes of Western Slopes Rosella result from hybridization between Crimson Rosella and Yellow Rosella in a species complex of Australian parrots. Grant, Grant, and Deutsch (1996) supported that hybridization can contribute to the speciation process by enhancing genetic variation and relaxing genetic constrains on particular directions of evolutionary change, such as some island birds (Darwin's Finches). In conclusion, the genetic exchange between the backcross offspring and *T. himalayensis* was weakened as a result that the mountains uplift promoted the ancestor of *T. altaicus* to colonize and Middle‐Asia aridification caused geographical barriers, and the interaction of these factors promoted a new species differentiate—*T. altaicus* (Figure 6).

## 5 CONCLUSIONS

The extant snowcocks originated from the QTP, and the uplift of the QTP and the Quaternary glacial oscillations accelerated the genus *Tetraogallus* diversification. Specifically, mountains uplift and competitive exclusion promoted the differentiation between *T. tibetanus* and *T. himalayensis*. The *T. himalayensis* colonized other suitable habitats during the glacial period, and the interglacial isolation promoted the speciation of the *T. caspius*. The hybridization between *T. tibetanus* and *T. himalayensis* reproduced fertile hybrids during the Quaternary glacial period and repeatedly backcrosses with *T. himalayensis* during the interglacial period as a result of inheriting many characteristics from *T. himalayensis*, and glacial dispersal and isolation finally promoted the speciation of *T. altaicus*.

## CONFLICT OF INTEREST

The authors declare that they have no conflict of interests.

## AUTHOR CONTRIBUTIONS

NF L conceived the study; L D was responsible for obtaining and analyzing data and wrote the manuscript with the help of JC L.

## Data Availability

All data used in this review paper have been published elsewhere. Genbank accession number please see the Tables A1 and A2.

## APPENDIX A

**Table A1:** GenBank accession number for mitochondrial and nuclear DNA sequences used in phylogenetic reconstructions for the *Tetraogallus* (4 ingroup and 2 outgroup taxa)

Taxa	Species	Mitochondrial (Coverage (%))					Nuclear (Coverage (%))			
		12S (100)	COX1 (83)	CYTB (100)	ND2 (83)	D‐loop (67)	CLTC (83)	CLTCL1 (83)	RHO (83)	EEF2 (83)
Ingroup	*T. tibetanus*	NC_023939.1	NC_023939.1	NC_023939.1	NC_023939.1	NC_023939.1	KC778959.1	KC778851.1	KC778916.1	KC778873.1
	*T. himalayensis*	NC_027279.1	NC_027279.1	NC_027279.1	NC_027279.1	NC_027279.1	KC785632.1	KC785646.1	KC785713.1	KC785663.1
	*T. caspius*	KJ001802.1	–	EU106676.1	–	–	–	–	–	–
	*T. altaicus*	KC785617.1	GQ482760.1	AY563127.1	KC785695.1	–	KC785631.1	KC785645.1	KC785712.1	KC785662.1
Outgroup	*A. chukar*	FJ752426.1	FJ752426.1	FJ752426.1	FJ752426.1	FJ752426.1	KC749574.1	KC749619.1	EF569435.1	KC749686.1
	*A. rufa*	KC749448.1	GU951807.1	HG940463.1	DQ307002.1	AJ586226.1	KC749576.1	KC749621.1	EF569436.1	KC749688.1

Note

12S, 12S ribosomal RNA; COX1, cytochrome c oxidase subunits 1; CYTB, cytochrome b; ND2, NADH dehydrogenase subunits 2; D‐loop, control region; CLTC, clathrin heavy polypeptide gene, intron 7; CLTCL1, clathrin heavy chain‐like 1 gene, intron 7; RHO, rhodopsin gene, intron 1; EEF2, eukaryotic elongation factor 2 (EEF2) gene, introns 5, 6 and partial coding sequence.

**Table A2:** Sequences information (D‐loop) of *T. tibetanus* and *T. himalayensis*

*T. tibetanus*			*T. himalayensis*		
Sampling site (size)	Haplotype	GenBank accession number	Sampling site (size)	Haplotype	GenBank accession number
QLS (1), TGL (5)	H1	JX136799.1	DLH (2)	H1	GQ343513.1
QLS (4)	H2	JX136800.1	GEM (2)	H2	GQ343514.1
QLS (2)	H3	JX136801.1	DLH (1)	H3	GQ343515.1
TGL (1)	H4	JX136802.1	SB (1)	H4	GQ343516.1
TGL (1)	H5	JX136803.1	SB (5)	H5	GQ343517.1
TGL (1)	H6	JX136804.1	SB (1)	H6	GQ343518.1
BKL (1)	H7	JX136805.1	SB (3)	H7	GQ343519.1
QLS (4), TGL (6), BKL (8), QDM (1)	H8	JX136806.1	DLH (2), KAS (3)	H8	GQ343520.1
TGL (1)	H9	JX136807.1	PS (3), ZS (2), AKS (1)	H9	GQ343521.1
QLS (1), TGL (1)	H10	JX136808.1	KAS (1)	H10	GQ343522.1
QLS (1), TGL (1), QDM (3)	H11	JX136809.1	PS (3)	H11	GQ343523.1
TGL (1)	H12	JX136810.1	KAS (1)	H12	GQ343524.1
QLS (1)	H13	JX136811.1	ZS (2)	H13	GQ343525.1
TGL (1)	H14	JX136812.1	AKS (1)	H14	GQ343526.1
TGL (1)	H15	JX136813.1	QT (3)	H15	GQ343527.1
TGL (1)	H16	JX136814.1	AKS (2)	H16	GQ343528.1
TGL (1), QDM (1)	H17	JX136815.1	HT (1)	H17	GQ343529.1
TGL (1)	H18	JX136816.1	KAS (1)	H18	GQ343530.1
TGL (1)	H19	JX136817.1	KAS (1)	H19	GQ343531.1
QLS (1)	H20	JX136818.1	KAS (2)	H20	GQ343532.1
TGL (4), QDM (1)	H21	JX136819.1	KAS (1)	H21	GQ343533.1
TGL (1)	H22	JX136820.1	QT (2)	H22	GQ343534.1
BKL (1)	H23	JX136821.1	KAS (3)	H23	GQ343535.1
BKL (1)	H24	JX136822.1	QT (1)	H24	GQ343536.1
KLS (1)	H25	JX136823.1	QT (1)	H25	GQ343537.1
KLS (1)	H26	JX136824.1	QT (1)	H26	GQ343538.1
KLS (1)	H27	JX136825.1	QT (1)	H27	GQ343539.1
QLS (1), QDM (1)	H28	JX136826.1	QT (1)	H28	GQ343540.1
QDM (2)	H29	JX136827.1	KAS (1)	H29	GQ343541.1
QDM (2)	H30	JX136828.1	KAS (1)	H30	GQ343542.1
QDM (1)	H31	JX136829.1	AKS (2)	H31	GQ343543.1
QDM (1)	H32	JX136830.1	TSK (2)	H32	GQ343544.1
QDM (1)	H33	JX136831.1	TSK (2)	H33	GQ343545.1
QLS (3)	H34	JX136832.1	TSK (1)	H34	GQ343546.1
QLS (1)	H35	JX136833.1	HT (1), SB (1)	H35	GQ343547.1
			HT (1)	H36	GQ343548.1
			HX (1)	H37	GQ343549.1

Note

Sampling sites of *T. tibetanus*: Qilian Mountains (QLS), Qaidam Basin (QDM), Baryan Har Mountains (BKL), Tanggula Mountains (TGL) and West Kunlun Mountains (WKL); Sampling sites of *T. himalayensis*: Delingha (DLH), Geermu (GEM), Subei (SB), Taxkorgan (TSK), Pishan (PS), Hotan (HT), Kashitashi (KAS), Aksu (AKS), Zhaosu (ZS), Qitai (QT) and Houxia (HX) (Please refer to the original for detailed sampling information; An et al., 2015; Wang et al., 2011).
